# Serum Biomarkers AFP, CEA and CA19-9 Combined Detection for Early Diagnosis of Hepatocellular Carcinoma

**Published:** 2019-02

**Authors:** Muhammad Ibrahim Alhadi EDOO, Vikram Kumar Chutturghoon, Gyabaah Kwabena WUSU-ANSAH, Hai ZHU, Tao Yang ZHEN, Hai Yang XIE, Shu-Sen ZHENG

**Affiliations:** 1. Division of Hepatobiliary Pancreatic Surgery, First Affiliated Hospital, School of Medicine, Zhejiang University, Hangzhou 310003, China; 2. Key Laboratory of Combined Multi-organ Transplantation, Ministry of Public Health, Hangzhou 310003, China

**Keywords:** Hepatocellular carcinoma, Primary hepatic cancer, Serum Biomarkers, Alpha fetoprotein, Carcinoembryonic antigen, Cancer antigen 199

## Abstract

**Background:**

We aimed to evaluate the whether AFP levels alone is an adequate screening indicator, or a combination of Generally, alpha-fetoprotein (AFP), CA19-9 and CEA could provide a better diagnostic tool in detecting and screening asymptomatic patients with primary hepatic cancer (PHC), and also evaluate the correlation of degree of differentiation with serum biomarker levels.

**Methods:**

We retrospectively reviewed the medical records of 1362 patients form 2014–2018 who visited the first Affiliated Hospital of Zhejiang University, Hangzhou, China for health check-ups or were diagnosed with cancer or cirrhosis. We then analyzed preoperative tumor markers level of AFP, CA19-9, and CEA. The standard reference values (AFP ≤20 ng/L CEA ≤ 5 ng/L, and CA19-9 ≤ 37 U/mL) were as positive or negative cut off values. Further, the histological sections of patients were categorized and correlated them with the three serum biomarkers.

**Results:**

Serum AFP, CEA, and CA19-9 levels in the PHC group were significantly higher compared to those with liver cirrhosis and healthy control groups (*P <* 0.03). With AFP as a single tumor marker for PHC diagnosis, it had a sensitivity of 63.3% with a specificity of 80.8%. AFP combined with CA19-9 and CEA showed specificity of 100%, a sensitivity 2.5% with the positive and negative predictive values of 100% and 22% respectively. Furthermore, histological evaluation revealed the highest AFP level of 9366.14±23902.61 ng/L associated with poorly differentiated HCC, while well-differentiated HCC, had the lowest mean AFP level of 45.19±181.27 ng/L.

**Conclusion:**

Combined serum levels of AFP, CA19-9 and CEA does not provide a superior advantage over AFP alone as a screening and diagnostic tool for HCC detection.

## Introduction

Hepatocellular carcinoma (HCC) is a primary malignancy of the liver that is associated with an increasing incidence and mortality rate. With annually reported cases, it is ranked the fifth most common and the second leading cause of cancer-related deaths worldwide. Globally, 800,000 new cases of HCC are reported annually, with more than 50% of cases occurring in China (1).

In mainland China, HCC is most prevalent among males, with an incidence of 58/100,000 compared to 53/100,000 in Taiwan and 29.9/100,000 in Hong Kong (2, 3). Currently, it is the second and third leading cause of cancer-related deaths in males and females respectively in China (4, 5). This rise in incidence over the past few decades has been attributed to high prevalence of chronic hepatitis B virus (HBV) infection (6–7). Over 93 million HBV carriers are Chinese, accounting for 2/3 of patients worldwide (8, 9). Currently, the standard treatment for HCC is comprehensive therapy predominantly in the form of surgery; resection and liver transplantation (7, 8). However, diagnosis is often made patients at a later or an advanced stage of the disease due to the non-specific symptomatic nature or the disease course, making surgical option less available to most patients. Hence, for decades, the prognosis of HCC patients has generally been poor (9–11). Following diagnosis, the median survival is approximately 6 to 20 months (12). The overall 5-year survival rate in patients with HCC who receive liver resection is about 40% compared to. 60–70% 5-year survival rate in early diagnosed HCC (9, 12). Often, one or more imaging modalities are often required for definitive diagnosis (13, 14). Ideally, tumors of approximately 2 cm in size without vascular or nodal invasion offer a better prognosis, while large tumor size with vascular invasion, poor functional status, and nodal metastases are associated with poor outcomes (15, 16).

The American Association for the Study of Liver Disease (AASLD), the National Comprehensive Cancer Network (NCCN), and the Asian Pacific Association for the Study of the Liver (APASL treatment guidelines recommend screening and surveillance as early detection of HCC currently is the most important predictor of treatment options and prognosis (17). Diagnostic imaging techniques include ultrasonography, computed tomography (CT), and magnetic resonance imaging (MRI) have proven to be. A systematic review has shown that ultrasonography has a sensitivity of 58% and a specificity of 94%, CT has shown sensitivity of 68% and a specificity of 93% while MRI has shown sensitivity of 81% and a specificity of 85% (18). Ultrasound, because of its simplicity, low cost, minimal invasiveness, and the fact that it allows real-time observation features, is the most common imaging tool used to screen for HCC. However, successful ultrasound detection relies on the expertise of the physician, the availability of ultrasound equipment, and the echogenicity of the liver. Thus, evaluating the actual sensitivity and specificity of ultrasound detection is difficult because of the lack of standards (19, 20). Serum biomarkers are striking potential tools to screen for and diagnose HCC early thanks to the non-invasive, objective, and reproducible assessments they can potentially enable.

Serum alpha-fetoprotein (AFP), cancer antigens (CA19-9), and carcinoembryonic antigen (CEA) levels were in patients with primary hepatic cancer (PHC), Liver cirrhosis, and the healthy population can be used as a screening and prognostic tool. In this study, we evaluated AFP, CA19-9 and CEA levels as well as positive rates, sensitivity and specificity of the serum markers in diagnosing hepatocellular carcinoma to establish a potential prognostic correlation between their serum levels.

## Methods

### Patient inclusion criteria

The study included a total of 1,362 subjects that were admitted for treatment or that had their physical check-up in the First Affiliated Hospital of Zhejiang University (Hangzhou, China) from 2014 to 2018. Totally 1075 subjects were patients with primary hepatic cancer (PHC). Out of these 1075 PHC study subjects, 909 were male and 166 were female with an age range of 18 to 91 yr, with an average of 56.2±11.0 years. 237 patients were diagnosed with liver cirrhosis, they were comprised of 187 men and 50 women, with a mean age of 49.31 ± 13 yr (range: 2–74 yr). 50 healthy individuals who underwent physical examinations in the same hospital were also included in this study. They served as a control in this study and were comprised of 36 men and 14 women, with a mean age of 61 ± 6 years (range: 40–75 yr). Patients enrolled in the study were chosen on the basis that they had no other underlying diseases or conditions and that no study subject was receiving chemotherapy or radiotherapy.

The data were retrieved from the Department of Hepatobiliary and Pancreatic Surgery, First Affiliated Hospital, School of Medicine, Zhejiang University. The study was reviewed and approved by the Institutional Review Board of First Affiliated Hospital, School of Medicine, Zhejiang University.

### Study design

A retrospective analysis of patients diagnosed with primary hepatic cancer and liver cirrhosis was collected from the hospital database. The patients enrolled in this study were chosen according to predefined inclusion criteria such as confirmed pathological diagnosis, all the 3 serum biomarkers under study, preoperative serum biomarkers level. Subjects major underlying diseases, metastatic history, or who had undergone treatment such as radiotherapy, chemotherapy, or endocrine therapy was excluded. Patients who fulfilled the criteria mentioned above were selected for the study. The primary hepatic cancer patients and the liver cirrhosis patients were compared to a group of healthy individuals from the same period as mentioned above.

In this study, 1075 primary hepatic cancer patients were selected and represented the primary hepatic cancer group (Group A), 237 liver cirrhosis patients were selected and represented the Cirrhosis group (Group B) and 50 healthy individuals were selected as control (Group C). After patients were grouped, we analyzed preoperative tumor markers level of these three different biomarkers AFP, CA19-9, and CEA. The normal reference values used for the three different biomarkers under study were as follows: AFP ≤20 ng/L CEA ≤ 5 ng/L, and CA19-9 ≤ 37 U/Mr. Patients with serum AFP > 20 ng/L, serum CA 19-9 >37 U/mL and a serum CEA >5ng/L were considered positive. The mean levels of all three different biomarkers in all Groups were calculated and the results were tabulated in Table 1.

**Table 1:** Serum AFP, CEA, and CA 19-9 levels compared between the three groups: primary hepatic cancer group (group A), cirrhotic group (group B) and the control group (group C)

***Group***	***N***	***AFP(ng/L)***	***CA19-9(U/mL)***	***CEA(ng/L)***
A	1075	4336.47±16094.35	40.90±342.38	3.11±3.76
B	237	28.41±73.17	23.58±33.34	2.76±1.78
C	50	<20.00	20.42±15.56	2.63±1.71

Group A= primary hepatic cancer, Group B: Cirrhosis, Group C= Control Results are mean ±SD

Additionally, serum biomarkers were combined as AFP and CA19-9; AFP and CEA; and AFP and CA19-9 and CEA, and the positive rates of the different combination of biomarkers in the three different groups were calculated as shown in Table 2.

**Table 2:** Positive rates of AFP, CEA and CA 19-9 combinations in the different study groups n (%)

***Serum markers***	***PHC (n=1075)***	***Cirrhosis (n=237)***	***Control (n=50)***
AFP	681(63.35)	53(22.3)	2(4)
AFP+CEA	83(7.7)	6(2.5)	1(2)
AFP+CA19-9	78(7.3)	15(6.8)	1(2)
AFP+CEA+CA19-9	27(2.5)	0(0)	0(0)

n=Number of positive cases

**Fig. 1:**
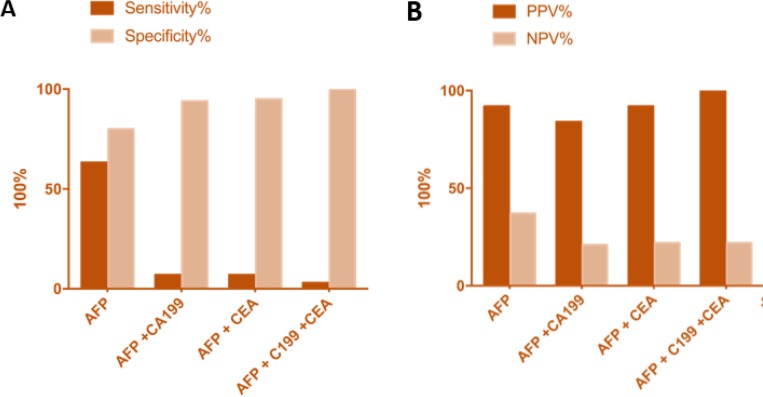
Sensitivity and specificity compared amongst AFP, CA19-9, CEA and combined analysis of all three biomarkers (**A**). positive and vegative predictive values of all three biomarkers (**B**)

The sensitivity, specificity, positive predictive values (PPV) and negative predictive values (NPV) were calculated for the different groups (Table 3) and Fig. 1.

**Table 3:** The sensitivities, specificities, Positive and Negative values compared between serum biomarkers AFP, and the different combination of AFP + CA19-9, AFP + CEA and AFP + CA19-9 + CEA

***Serum biomarkers***	***Sensitivity %***	***Specificity %***	***PPV%***	***NPV%***
AFP	63.3	80.8	92	37
AFP + CA19-9	7.3	94.42	84	21
AFP + CEA	7.6	95.56	92	22
AFP+CA19-9+CEA	2.5	100	100	22

The patients in the primary hepatic cancer group, group A, were further divided according to their pathological types as follows: poorly differentiated hepatocellular carcinoma (104 patients); moderately-poorly differentiated hepatocellular carcinoma (412 patients); moderately differentiated hepatocellular carcinoma (422 patients); well-moderately differentiated hepatocellular carcinoma with 45 study subjects; Well-differentiated hepatocellular carcinoma with 27 study subjects; and combined hepato-cholangiocarcinoma with 65 study subjects. Their mean serum biomarkers level for all the three biomarkers were calculated and the positive rates of AFP combined with CA19-9 and CEA were calculated in the different groups.

### Statistical analysis

Statistical analysis was performed using SPSS version 18.0 statistical software (SPSS Inc., Chicago, IL, United States). The data were expressed as mean ± SD. Measurement data between groups were compared with the *t*- distribution. All test was two-tailed and a *P*-value of less than 0.05 was considered statistically significant.

## Results

### The significance of AFP CA19-9 and CEA levels among groups

Significant differences in mean serum levels and positive rate of AFP, CEA and CA19-9 were observed between the PHC group and the other two groups (*P*< 0.03). There were no significant differences between the control and benign liver cirrhosis groups as shown in Tables 3–4. In the primary hepatic cancer group (group A), an increased AFP level above the cut off level was observed in 681 study subjects accounting for 63.35% of the PHC patients; 83 subjects had increased AFP and CEA levels (7.7%); 78 subjects had increased AFP and CA19-9 levels (7.3%); and 27 subjects had increased levels of AFP, CA19-9 and CEA (2.5%). The mean serum marker level for AFP was 4336.47±16094.35 ng/L, 40.90±342.38 U/mL for CA19-9 and 3.11±3.76 ng/L for CEA in the HCC group. In the Liver cirrhosis group (group B), 53 out of the 237 patients tested positive for elevated AFP (22.4%); 6 tested positive for AFP and CEA (2.5%); 15 tested positive for AFP and CA19-9 (6.8%); and none tested positive for AFP, CA19-9 and CEA (0%). The mean AFP level was 28.41±73.17ng/L, mean CA19-9 level was 23.58±33.34 U/mL and the mean CEA level was 2.76±1.78 ng/L. In the control group, 2 out of the 50 patients tested positive for AFP (4%); 1 positive for AFP and CEA (2%); 1 positive for AFP and CA19-9 (2%); and none tested positive for AFP, CEA, and CA19-9. The mean AFP level was <20.00μg/L, mean CA19-9 level was 20.42±15.56 U/mL and the mean CEA level was 2.63±1.71 ng/L. The mean serum AFP, CA19-9 and CEA levels were clearly higher in the PHC group than in the other liver cirrhosis and control groups.

**Table 4:** Comparable serum levels of AFP, CA19-9 and combination of AFP+CEA+CA19-9 in relation to the different pathological types (Poorly diff, Moderately-poorly diff, Moderately diff, Well-moderately diff, Well diff and Combined Hepato-Cholangiocarcinoma) of HCC

***Diagnosis***	***N***	***AFP (ng/L)***	***CEA (ng/L)***	***CA19-9 (U/mL)***	***AFP+CEA+CA19-9(n)***
Poorly dif HCC	104	9366.14±23902.61	2.99±2.32	43.35±206.86	1
Moderately-poorly dif HCC	412	8170.17±20781.66	3.00±4.63	53.27±318.64	10
Moderately dif HCC	422	1686.38±7922.22	2.95±1.89	20.94±58.03	13
Well- moderately dif HCC	45	164.20±666.20	3.78±2.13	21.03±23.82	1
Well dif HCC	27	45.19± 181.27	2.45± 1.41	13.60±13.92	0
Combined Hepato-Cholangiocarcinoma	65	2134.73±6158.24	4.51±7.83	113.29±356.71	2

N=Number of studied subjects; n=number of positive cases of AFP+CA19-9+CEA dif=differentiated

### AFP levels increase significantly in primary liver cancer compared to other markers

Comparing serum biomarkers levels in the primary hepatic cancer group (group A) to the cirrhotic group (group B) and the control group (group C), significant statistical differences (*P* < 0.03 were observed between the malignant group and the benign group, whereas no statistically significant difference (*P* > 0.06) were present between the Liver cirrhosis group and the healthy control group, thus indicating higher serum AFP, CA19-9 and CEA mean levels in the cancer group as compared to the other two groups. Moreover, a higher positive rate of the different combinations of biomarkers was observed in the PHC group compared with the other two groups. The mean serum marker level for AFP in the cancer group was 4336.47±16094.35 ng/L as compared to 28.41±73.17μg/L in the Liver cirrhosis group and <20.00μg/L in the healthy control group. This shows that AFP serum level had greatly increased in the cancer group by 154-fold as compared to the liver cirrhosis group. The mean serum marker level for CA19-9 in the cancer group was 40.90±342.38 U/mL as compared to 23.58±33.34 U/mL in the Liver cirrhosis group and 20.42±15.56 U/mL in the healthy control group. This shows that CA19-9 serum level had a considerable increased in the cancer group by a 2-fold as compared to the liver cirrhosis group and the control group. The mean serum marker level for CEA in the cancer group was 3.11±3.76 ng/L as compared to 2.76±1.78 ng/L U/mL in the Liver cirrhosis group and 2.63±1.71 ng/L in the healthy control group. This shows a 1.2-fold increase in serum level CEA in the cancer group compared to the liver cirrhosis group and the control group. Thus, this finding indicates increased serum AFP levels in the Primary Hepatic Cancer patients compared to the other 2 groups. Also, a slight increase in serum CA19-9 and CEA levels were noted compared to a large increase observed with AFP levels.

### AFP compares to combination markers as a stand-alone marker

AFP, as a stand-alone marker, showed a sensitivity of 63.3%, a specificity of 80.8% with a 92% PPV and a 37% NPV. Combining AFP with CA19-9, a sensitivity of 7.3%, specificity of 94.42% with 84% PPV and a 21% NPV was observed. The combination with AFP with CEA showed sensitivity of 7.6%, specificity of 95.56% with 92% PPV and a 22% NPV. The combination of all three markers AFP CA19-9 and CEA showed sensitivity of 2.5%, specificity of 100% with 100% PPV and a 22% NPV. Though a higher specificity was observed with combined AFP, CA19-9 and CEA (100%) with high Positive predictive value (100%), the sensitivity was very low (2.5 %) compared to the sensitivity of AFP alone. The lower sensitivity observed in the combinations of the three serum biomarkers prevents them from been used a potential diagnostic tool and thus has no superior advantage as a diagnostic and screening tool than AFP alone.

### Combined AFP, CA19-9, and CEA has increased specificity but decreased sensitivity.

The sensitivities and specificities of the different combinations of the serum biomarkers, as well as their positive predictive values (PPV) and negative predictive values (NPV), were computed as shown in Table 3. The sensitivities of AFP; AFP and CA19-9; AFP and CEA; and AFP, CA19-9 and CEA were 63.3%, 7.3%, 7.6% and 2.5% respectively. The sensitivity of AFP alone was greater than the sensitivity of AFP combined with the other two biomarkers. The specificities of AFP; AFP and CA19-9; AFP and CEA; and AFP, CA19-9, and CEA were 80.8%, 94.4%, 97.6% and 100% respectively. The specificity of AFP combined with CA19-9; CEA; CA19-9 and CEA were higher than the specificity of AFP alone. The PPV of AFP; AFP and CA19-9; AFP and CEA; and AFP, CA19-9 and CEA were 92%, 84%, 92% and 100% respectively. The NPV of AFP; AFP and CA19-9; AFP and CEA; and AFP, CA19-9 and CEA were 37%, 21%, 22% and 22% respectively.

### Pathological differentiation correlates with significantly increased AFP levels but not CEA and CA19-9 levels

To assess the correlation between serum markers and degree of differentiation, 104 patients diagnosed with poorly differentiated HCC had a mean AFP level of 9366.14±23902.61 ng/L, mean CA19-9 level of 43.35±206.86 U/mL and mean CEA level of 2.99±2.32 ng/L and 1 patient out of the 104 was positive for all the 3 biomarkers. Of the patients, diagnosed with moderate-poorly differentiated HCC, 412 patients had a mean AFP level of 8170.17±20781.66 ng/L, mean CA19-9 level of 53.27±318.64 U/mL and mean CEA level of 3.00±4.63 ng/L and ten patients out of the 412 tested positive for all the three biomarkers. Amongst the patients diagnosed with moderate-poorly differentiated HCC 422 of them had a mean AFP level of 1686.38±7922.22 ng/L, mean CA19-9 level of 20.94±58.03 U/mL and mean CEA level of 2.95±1.89 ng/L and 13 patients out of the 422 was positive for all the three biomarkers. Of those diagnosed with moderate-poorly differentiated HCC, 45 patients had a mean AFP level of 164.20±666.20 ng/L, mean CA19-9 level of 21.03±23.82 U/mL and mean CEA level of 3.78±2.13 ng/L with 1 patient testing positive for all the three biomarkers. All 27 patients diagnosed with well-differentiated HCC, had a mean AFP level of 45.19±181.27 ng/L, mean CA19-9 level of 13.60±13.92 U/mL and mean CEA level of 2.45±1.41 ng/L with no patient testing positive for all the three biomarkers. Out of the patients diagnosed with moderately-poor differentiated HCC, 65 had a mean AFP level of 2134.73±6158.24 ng/L, mean CA19-9 level of 113.29±356.71 U/mL and mean CEA level of 4.51±7.83 ng/L and two patients only were positive for all the three biomarkers, as shown in (Table 4).

## Discussion

HCC is one of the most common malignant tumors. Early diagnosis and early surgical resections are imperative for improving the survival of HCC patients. The incidence of hepatocellular carcinoma has increased worldwide as well as in China in the recent decade. Its prevalence has been increased mostly due to an increase in the rate of HBV infections (21–23). AFP, a specific glycoprotein produced primarily by the fetal liver has been the most practical and widely used serum biomarker for HCC diagnosis. However, its sensitivity and specificity vary significantly from 40%–65% and 76%–96%, respectively (23–25). This has increased the demand for specific biomarkers that can lead to the early diagnosis and improved prognosis. In this study, we systematically evaluated the role of combining serum levels of CA19-9 and CEA to AFP in diagnosing hepatocellular carcinoma. Alpha-fetoprotein (AFP), a fetal-specific glyco-protein antigen, is the most commonly used serological biomarker and is considered as a useful and practical tool for the screening and early diagnosis of HCC in clinical practice. However, the clinical diagnostic accuracy of AFP is unsatisfactory due to the wide variation in its sensitivity and specificity observed making elevated AFP non-specific, especially in the early stages of HCC. AFP has been found to have a sensitivity of 39–65% and a specificity of 76–94% in detecting HCC AFP cut-off value of 20ng/mL (26).

However, in up to 30% of patients with HCC, AFP levels are underexpressed and goes undetected as AFP levels fall within the normal range (27). Moreover, overexpression of AFP levels can also be observed in some patients with the non-malignant chronic liver disease, including 15–58% with chronic hepatitis and 11–47% with liver cirrhosis (28). These variations in AFP levels observed in both malignant and benign patients presents a diagnostic challenge in some cases as a screening tool in diagnosing HCC. This has opened up a potential research field to detect biomarkers to complement AFP to achieve early diagnosis and better prognosis.

In the current study, we found a higher prevalence of moderately differentiated HCC (39.3 %) compared to poorly differentiated HCC (9.7%), moderate-poorly differentiated HCC (38.3%), Well-moderately differentiated HCC (4.2%), well-differentiated HCC (2.5%), and Combined Hepato-Cholangiocarcinoma (6.1%). The rates of AFP+CA19-9+CEA being positive were 3.7% (1/27), 37% (10/27), 3.7% (1/27), 0% (0/27) and 7.4% (2/27) respectively.

Furthermore, we observed that patients with poorly differentiated HCC expressed significantly increased AFP level of 9300+ ng/L, while well-differentiated HCC pathology expressed very low serum AFP levels. The most observed diagnosis was that of moderately differentiated HCC with 422 study subjects diagnosed (422/1075, 39.3%) and had the higher positive rates of AFP+CA19-9+CEA (13/27, 48%). The least observed diagnosis was that of well-differentiated HCC with 27 study subjects (27/1075, 2.5%) and had no positive rates of AFP+CA19-9+CEA (Table 4). Based on our results we found that in well-differentiated HCC AFP levels approximate to a level of about <200 ng/L; in well-moderately differentiated HCC AFP levels are observed at about 200–1600 ng/L; in moderately differentiated HCC AFP levels were about 2000 ng/L; in moderately-poorly differentiated HCC, AFP levels were recorded at 2000–8000ng/l; and in poorly differentiated HCC AFP levels were > 8000ng/L. The combined AFP+CA19-9+CEA or AFP+CA19-9 or AFP+CEA although have low sensitivity, it high specificity makes it a better marker to rule out HCC when patients test, making it a potential definitive and differential diagnostic combined marker.

## Conclusion

Although AFP combined with CA19-9 and CEA, has a specificity of 100% and a positive predictive value of 100% its low sensitivity of 2.5 % makes it use as a screening tool inferior to AFP alone in HCC and differentiating HCC from non-HCC patients, and therefore not a suitable substitute in screening of potential HCC patients, however it can aid in the definitive diagnosis of HCC and exclude HCC as the primary. In summary, although we propose the combination of AFP, CA19-9 and CEA for HCC surveillance in HCC patients, the search for novel biomarkers of early HCC detection requires further research.

## Ethical considerations

Ethical issues (Including plagiarism, informed consent, misconduct, data fabrication and/or falsification, double publication and/or submission, redundancy, etc.) have been completely observed by the authors.

## References

[1] Torre LA Bray F Siegel RL Ferlay J Lortet-Tieulent J Jemal A (2015). Global cancer statistics, 2012. CA Cancer J Clin, 65:87–108. 25651787 10.3322/caac.21262

[2] Yuen MF Hou JL Chutaputti A (2009). Hepatocellular carcinoma in the Asia Pacific region. J Gastroenterol Hepatol, 24:346–353. 19220670 10.1111/j.1440-1746.2009.05784.x

[3] Asia-Pacific Working Party on Prevention of Hepatocellular Carcinoma (2010). Prevention of hepatocellular carcinoma in the Asia-Pacific region: consensus statements. J Gastroenterol Hepatol, 25:657–663. 20492323 10.1111/j.1440-1746.2009.06167.x

[4] Song P Feng X Zhang K (2013). Perspectives on using des-gamma-carboxyprothrombin (DCP) as a serum biomarker: facilitating early detection of hepatocellular carcinoma in China. Hepatobiliary Surg Nutr, 2:227–231. 24570947 10.3978/j.issn.2304-3881.2013.08.11 PMC3924680

[5] Chinese Anti-Cancer Association Society of Liver Cancer, Chinese Society of Clinical Oncology, Chinese Society of Hepatology Liver Cancer Study Group The expert consensus on the treatment standards for hepatocellular carcinoma. Digestive Disease and Endoscopy 2009. (in Chinese)

[6] Tanaka M Katayama F Kato H (2011). Hepatitis B and C virus infection and hepatocellular carcinoma in China: a review of epidemiology and control measures. J Epidemiol, 21:401–416. 22041528 10.2188/jea.JE20100190 PMC3899457

[7] Kudo M Han KH Kokudo N (2009). Liver Cancer Working Group report. Jpn J Clin Oncol, 40 Suppl 1:i19–27. 10.1093/jjco/hyq123 20870915

[8] Song P Feng X Zhang K (2013). Screening for and surveillance of high-risk patients with HBV-related chronic liver disease: promoting the early detection of hepatocellular carcinoma in China. Biosci Trends, 7:1–6. 23524887

[9] Zhang C Zhong Y Guo L (2013). Strategies to prevent hepatitis B virus infection in China: immunization, screening, and standard medical practices. Biosci Trends, 7:7–12. 23524888

[10] Oka H Tamori A Kuroki T Kobayashi K Yamamoto S (1994). Prospective study of alpha-fetoprotein in cirrhotic patients monitored for development of hepatocellular carcinoma. Hepatology, 19:61–66. 7506227

[11] Zoli M Magalotti D Bianchi G Gueli C Marchesini G Pisi E (1996). Efficacy of a surveillance program for early detection of hepatocellular carcinoma. Cancer, 78:977–985. 8780534 10.1002/(SICI)1097-0142(19960901)78:5<977::AID-CNCR6>3.0.CO;2-9

[12] Anonymus (1998). A new prognostic system for hepatocellular carcinoma: a retrospective study of 435 patients: the Cancer of the Liver Italian Program (CLIP) investigators. Hepatology, 28:751–755. 9731568 10.1002/hep.510280322

[13] Siegel RL Miller KD Jemal A (2018). Cancer statistics 2018. CA Cancer J Clin, 68(1):7–30. 29313949 10.3322/caac.21442

[14] Forner A Llovet JM Bruix J (2012). Hepatocellular carcinoma. Lancet, 379:1245–1255. 22353262 10.1016/S0140-6736(11)61347-0 PMC13537732

[15] Bruix J Sherman M Practice Guidelines Committee, American Association for the Study of Liver Diseases (2005). Management of hepatocellular carcinoma. Hepatology, 42:1208–1236. 16250051 10.1002/hep.20933

[16] Llovet JM Bustamante J Castells A (1999). Natural history of untreated nonsurgical hepatocellular carcinoma: rationale for the design and evaluation of therapeutic trials. Hepatology, 29:62–67. 9862851 10.1002/hep.510290145

[17] Song P Tobe RG Inagaki Y (2012). The management of hepatocellular carcinoma around the world: a comparison of guidelines from 2001 to 2011. Liver Int, 32:1053–1063. 22432445 10.1111/j.1478-3231.2012.02792.x

[18] Song P Gao J Inagaki Y (2013). Biomarkers: Evaluation of Screening for and Early Diagnosis of Hepatocellular Carcinoma in Japan and China. Liver Cancer, 2(1):31–9. 24159594 10.1159/000346220 PMC3747538

[19] Aghoram R Cai P Dickinson JA (2012). Alpha-foetoprotein and/or liver ultrasonography for screening of hepatocellular carcinoma in patients with chronic hepatitis B. Cochrane Database Syst Rev, Cd002799. 22972059 10.1002/14651858.CD002799.pub2 PMC6464874

[20] Amarapurkar D Han KH Chan HL Ueno Y Asia-Pacific Working Party on Prevention of Hepatocellular Carcinoma (2009). Application of surveillance programs for hepatocellular carcinoma in the Asia-Pacific Region. J Gastroenterol Hepatol, 24:955–961. 19383082 10.1111/j.1440-1746.2009.05805.x

[21] El-Serag HB Kanwal F (2014). Epidemiology of hepatocellular carcinoma in the United States: where are we? Where do we go? Hepatology, 60:1767–1775. 24839253 10.1002/hep.27222 PMC4211957

[22] American Cancer Society (2015). Cancer Facts And Figures. Atlanta, Ga. American Cancer Society. https://www.cancer.org/content/dam/cancer-org/research/cancer-facts-and-statistics/annual-cancer-facts-and-figures/2019/cancer-facts-and-figures-2019.pdf

[23] Trevisani F D’Intino PE Morselli-Labate AM (2001). Serum alpha-fetoprotein for diagnosis of hepatocellular carcinoma in patients with chronic liver disease: influence of HBsAg and anti-HCV status. J Hepatol, 34:570–575. 11394657 10.1016/s0168-8278(00)00053-2

[24] Khien VV Mao HV Chinh TT (2001). Clinical evaluation of lentil lectin-reactive alpha-fetoprotein-L3 in histology-proven hepatocellular carcinoma. Int J Biol Markers, 16:105–111. 11471892 10.1177/172460080101600204

[25] Yoshida S Kurokohchi K Arima K (2002). Clinical significance of lens culinaris agglutinin-reactive fraction of serum alpha-fetoprotein in patients with hepatocellular carcinoma. Int J Oncol, 20:305–309. 11788893

[26] Daniele B Bencivenga A Megna AS Tinessa V (2004). Alpha-fetoprotein and ultrasonography screening for hepatocellular carcinoma. Gastroenterology, 127:S108–112. 15508073 10.1053/j.gastro.2004.09.023

[27] Farinati F Marino D De Giorgio M (2006). Diagnostic and prognostic role of alpha-fetoprotein in hepatocellular carcinoma: both or neither? Am J Gastroenterol, 101:524–532. 16542289 10.1111/j.1572-0241.2006.00443.x

[28] Johnson PJ (2001). The role of serum alpha-fetoprotein estimation in the diagnosis and management of hepatocellular carcinoma. Clin Liver Dis, 5:145–159. 11218912 10.1016/s1089-3261(05)70158-6

